# Intrinsically Disordered Linkers Impart Processivity on Enzymes by Spatial Confinement of Binding Domains

**DOI:** 10.3390/ijms20092119

**Published:** 2019-04-29

**Authors:** Beata Szabo, Tamas Horvath, Eva Schad, Nikoletta Murvai, Agnes Tantos, Lajos Kalmar, Lucía Beatriz Chemes, Kyou-Hoon Han, Peter Tompa

**Affiliations:** 1 Institute of Enzymology, Center of Natural Sciences, Hungarian Academy of Sciences, 1117 Budapest, Hungary; szabo.beata@ttk.mta.hu (B.S.); hotafin@gmail.com (T.H.); schad.eva@ttk.mta.hu (E.S.); murvai.nikoletta@ttk.mta.hu (N.M.); tantos.agnes@ttk.mta.hu (A.T.); lk397@cam.ac.uk (L.K.); 2 Instituto de Investigaciones Biotecnológicas IIB-INTECH, Consejo Nacional de Investigaciones Científicas y Técnicas (CONICET), Universidad Nacional de San Martín, Buenos Aires 1650, Argentina; lchemes@iibintech.com.ar; 3 Genome Editing Research Center, Division of Biomedical Science, Korea Research Institute of Bioscience and Biotechnology (KRIBB), Daejeon 34113, Korea; khhan600@kribb.re.kr; 4 Department of Nano and Bioinformatics, University of Science and Technology (UST), Daejeon 34113, Korea; 5 VIB Center for Structural Biology, Vrije Univresiteit Brussel, 1050 Belgium, Brussel

**Keywords:** enzyme efficiency, polymeric substrate, processive enzyme, disordered linker, binding motif, binding domain, spatial search, local effective concentration

## Abstract

(1) Background: Processivity is common among enzymes and mechanochemical motors that synthesize, degrade, modify or move along polymeric substrates, such as DNA, RNA, polysaccharides or proteins. Processive enzymes can make multiple rounds of modification without releasing the substrate/partner, making their operation extremely effective and economical. The molecular mechanism of processivity is rather well understood in cases when the enzyme structurally confines the substrate, such as the DNA replication factor PCNA, and also when ATP energy is used to confine the succession of molecular events, such as with mechanochemical motors. Processivity may also result from the kinetic bias of binding imposed by spatial confinement of two binding elements connected by an intrinsically disordered (ID) linker. (2) Method: By statistical physical modeling, we show that this arrangement results in processive systems, in which the linker ensures an optimized effective concentration around novel binding site(s), favoring rebinding over full release of the polymeric partner. (3) Results: By analyzing 12 such proteins, such as cellulase, and RNAse-H, we illustrate that in these proteins linker length and flexibility, and the kinetic parameters of binding elements, are fine-tuned for optimizing processivity. We also report a conservation of structural disorder, special amino acid composition of linkers, and the correlation of their length with step size. (4) Conclusion: These observations suggest a unique type of entropic chain function of ID proteins, that may impart functional advantages on diverse enzymes in a variety of biological contexts.

## 1. Introduction

Processivity is a kinetic phenomenon widespread among enzymes that act on polymeric substrates, such as DNA, RNA, polysaccharides, and proteins [1]. Once committed, processive enzymes engage in multiple rounds of modification instead of releasing their substrate after modifying it once. Served by different sliding mechanism(s), very effective enzymatic modifiers arose in evolution that can carry out hundreds or thousands of elementary steps upon a single engagement with the substrate [1]. Processivity occurs in: (i) synthesis (e.g., DNA by DNA polymerase [2], RNA by RNA polymerase, and protein by the ribosome [3]); (ii) degradation (e.g., DNA by DNAse [4], RNA by RNAse [5], polysaccharides by glycohydrolases [6] or proteins by the proteasome [7,8]); (iii) structural modification (e.g., DNA by helicase [9]); (iv) chemical modification (e.g., ubiquitination of proteins by ubiquitin ligases [10,11]); or (v) cargo transport (e.g., movement by mechanochemical motors kinesin, dynein and myosin [12,13,14,15] along actin and tubulin tracks).

A compilation of domain-linker-domain (DLD)-type monomeric processive enzymes is taken from the comprehensive list given in Supplementary Table S1. Important parameters including the length of predicted disordered linker, mean linker length of orthologous proteins (see Table S2 for species), κ value describing charge distribution, and the level of processivity (such as the length of processive move, the number of steps taken or the number of elementary substrate units covered, if determined at all), are given. 

**Figure 1:**
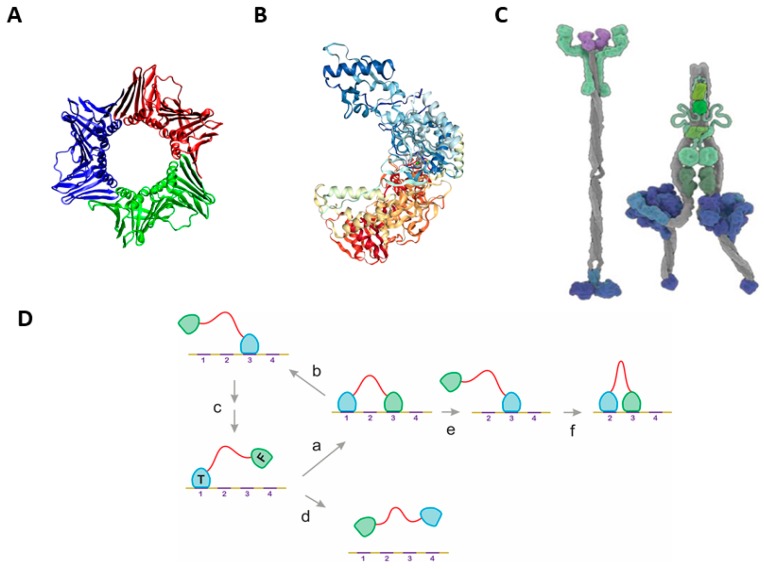
Basic mechanisms of processivity. The figure illustrates the two basic types (and four subtypes) of the mechanism of processivity. The classical mechanism based on structural confinement are represented by folded proteins that either (**A**) completely surround their partner by an oligomeric structure of toroidal shape, such as PCNA (PDB: 1AXC) [16,17], or (**B**) use an asymmetric binding domain to restrict its dissociation, such as in HIV reverse transcriptase (PDB: 1REV) [18]. Basically, different mechanisms are based on spatial confinement allowed by two binding motifs connected by a long, adaptable or flexible linker, as appears in (**C**) the ATP-dependent dimeric mechanochemical motors kinesin-1 and dynein (adapted from [20]), or (**D**) monomeric processive enzymes of domain-disordered linker-domain arrangement. These types of enzymes analyzed here in detail (for cases, see Table 1) bind their polymeric substrate via two binding domains, termed “bound” or “tethered” (T) for the one that anchors the enzyme to the substrate and “unbound” or “free” (F) for the one that is in search for substrate “target” binding sites), connected by a structurally disordered linker. We show by statistical-kinetic modeling that binding via the tethering domain kinetically favors binding via the free domain (a) over full dissociation of the protein (d), which may then result in processive diffusional moves (c) or directed movements driven by energy-dependent binding and/or modification of the substrate (e,f).

Given the extreme diversity of substrates upon which these processive enzymes act and also the variability of the chemical/mechanochemical changes they make, it is of little surprise that the molecular details of processivity are rather diverse, yet they are based on combinations of two basic designs principles. The classic and amply studied mechanism relies on structural confinement by circular/cylindrical or asymmetric binding domains or subunits of the enzymes. The former occurs, for example, when the PCNA subunit of DNA polymerase encircles the template DNA (Figure 1A) to ensure that the enzyme adds a practically unlimited number of nucleotides [16,17] to the growing DNA polymer. A closely related solution is used by HIV reverse transcriptase [18], which has an asymmetric binding domain that strongly favors sliding along the RNA substrate over dissociating from it (Figure 1B). A completely different mechanism has evolved in mechanochemical motors, such as kinesin and dynein, which move along polymeric protein tracks of tubulin [15]. These dimeric proteins have long coiled-coil stalks and ATPase binding domains, which undergo conformational changes that result in a strong preference for rebinding following dissociation due to a proximity effect, i.e., spatial confinement (Figure 1C). The region connecting the dimerization domain with the binding domain may even undergo transitions between ordered and disordered states [19]. The latter class of processive motors suggests that the presence of two binding elements (motifs or domains) connected by long, conformationally adaptable/flexible linker region(s) appears to be a key element of processivity, which combines deterministic and probabilistic elements of binding [20]. 

Here we generalize this concept by observing and analyzing that proteins in which binding domains are connected by a disordered linker may show probabilistic bias for re-binding over dissociation from their substrate, due to which they possess processive capacity. As structural disorder of proteins (intrinsically disordered protein/region, IDP/IDR) is widespread in eukaryotic proteomes [21,22], this may be a frequently applied mechanism. IDPs/IDRs often engage in protein-protein interactions [23,24] but their function may also directly stem from the disordered state, termed entropic-chain functions [25]. Binding and entropic-chain functions can actually be combined because often part of the IDP remains disordered even in the bound state, a phenomenon termed fuzziness [26]. Of particular relevance to the observed processivity is that binding motifs embedded in disordered regions, due to the arising “proximity effect” or “optimal effective concentration” around binding sites, may feature facilitated binding, which is central to the concepts of: (i) acceleration of binding by “fly casting” [27], (ii) reduction of binding dimensionality by the “monkey-bar” mechanism [28], and (iii) “ultrasensitive” binding by repetitive binding motifs in signaling proteins [29,30].

By statistical-physical modeling and bioinformatics analysis we show that this kinetic proximity effect is also a widespread inherent property of many monomeric processive enzymes that are capable of multiple rounds of modification of their polymeric substrate. These enzymes, such as a variety of glycohydrolases (e.g., cellulases) [6,31,32], Ribonuclease H1 (RNAse-H1) [5] and matrix metalloproteinase-9 (MMP-9) [33], need no ATP energy for processivity, which makes it a robust and widespread mechanism in the proteome. Here we have selected 12 such monomeric (ATP-independent) processive enzymes from the literature and provide a comprehensive analysis of their physical and structural properties. We show that once engaged with their substrate, their structural organization kinetically biases binding of their free binding domain over dissociation of both its domains, resulting in multiple successive binding events without ever fully releasing the polymeric partner (Figure 1D). We suggest that this type of processivity represents a unique type of “entropic chain” function enabled by the structural disorder of their linker region [25,34], which may be a general mechanism that arises in a broad range of biological contexts.

## 2. Results

### 2.1. The Classical Mechanisms of Processivity

For rationalizing the diverse mechanisms of processivity, we suggest that they fall into two broad mechanistic categories (cf. Table S1). The structural underpinning of the mechanism is straightforward when the enzyme uses structural confinement to make dissociation from the substrate highly unfavorable [1]. Complete confinement may result from ring-shaped oligomeric structures (e.g., PCNA [16,17] (Figure 1A)), whereas asymmetric structures of a single polypeptide chain can also either fully (e.g., exonuclease I [1]) or partially (e.g., HIV reverse transcriptase [18] (Figure 1B)) enclose the substrate. These mechanisms can be interpreted in terms of a preferred 1D sliding of the substrate (template) within the well-defined structural element of the enzyme. 

Processivity of a completely different structural rationale can be observed in motor enzymes that use chemical energy for unidirectional movement along cytoskeletal tracks [12,13]. These motors usually have a dimeric structure, with their dimerization region and ATPase domains connected to their substrate-binding domains by long and extended structures (stalk) (Figure 1C). Large-scale conformational changes elicited by ATP hydrolysis in the ATPase domain(s) propagate to these binding domains, which result in a preference for the re-binding to the substrate track vs. full dissociation [14,15]. In these mechanisms, passive diffusional moves and energy-driven directional steps are combined, i.e., they represent a combination of confining the sequence of events by structural and spatial means. As outlined in the next paragraph, confinement by the limitation of search space by a disordered linker connecting binding domains (Figure 1D) can also account for processivity of enzymes, which appears to be widely applied in biology.

### 2.2. Statistical Physical Modelling of Domain-Linker-Domain Enzymes

**Figure 2:**
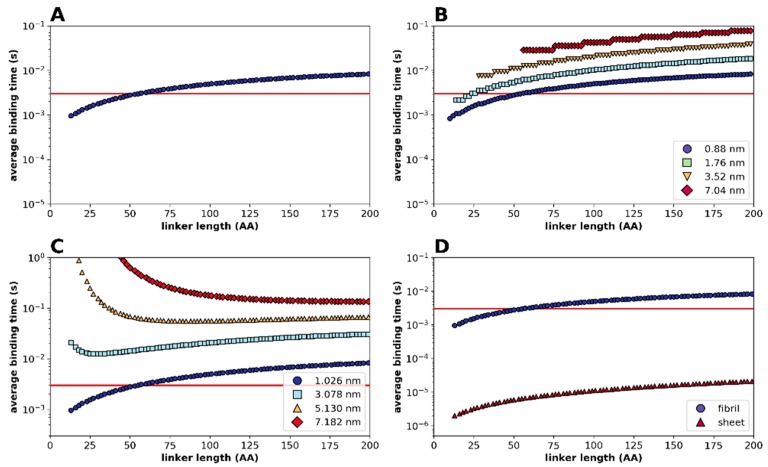
Modelling linker length in processive enzymes. Average binding times (tb) of a free domain linked to the tethering domain already bound to the substrate by a disordered linker of the given length (cf. Figure 1D, and Supplementary Equations (S9) and (S10)). The substrate is modelled based on cellulose geometry: it is assumed to contain binding sites spaced equidistantly every 1.026 nm (1 cellobiose unit) in the X dimension for a thread, and every 2 nm in the Y dimension in case of a sheet. (**A**) Average binding time of the free domain with a random-coil linker (length of Kuhn segment (lk) = 0.88 nm) and binding domains with no physical dimensions. (**B**) Lengthening the Kuhn segment length from 0.88 nm (random-coil) to 7.04 nm (PPII helix) significantly slows binding and reduces processivity. (**C**) “Diluting” binding sites on the substrate (by lengthening the distance between binding sites from 1 cellobiose unit to 7) has a dramatic effect on binding time. (**D**) Binding to a 2D substrate (sheet) is much faster than binding to a 1D substrate (fibril), making the enzyme more processive. On all the panels, if we assume a dissociation half-time of 3 × 10^−3^ s (limited by catalysis), the enzyme is typically processive at shorter, but not at longer, linker lengths (see text for details).

In order to determine how the disordered linker influences (re)binding kinetics of binding domains within a DLD-type enzyme, we used a statistical-kinetic approximation of their binding/unbinding behavior. As the effect of linker length will depend on distances between binding sites and on/off rates of binding domains, we used as a representative example the cellulose/cellulase (Cel7A in Table 1) system. To describe the kinetic behavior of the system, we used a Gaussian approximation of the exact Freely Jointed Chain (FJC) model (see Supplementary Methods and Figure S1). Figure 2 shows the results of varying parameters of a sample case where the tethering domain (cf. Figure 1D) is bound at a substrate site, and we calculate the average binding time (the time it takes for half the free domains to bind a target binding site on the substrate; cf. Supplementary Methods, Equations (S9) and (S10)). By considering the distribution of concentration of the free domain around the bound tethering domain (Figure S1) and integrating binding events (kinetics) based on the binding rate of cellulases (Table S3) over all binding sites within the reach of the free domain, it appears (Figure 2A) that the average time required for re-binding (Supplementary Equation (S10)) increases with increasing linker length. By assuming a threshold set by the kinetics of the dissociation of the tethering domain (for illustration, dissociation half-time (i.e., the time taken for half the bound domains to dissociate) taken as 3 × 10^−3^ s), the system is processive below a certain linker length (re-binding will be preferred over dissociation), and becomes non-processive for longer linkers (e.g., the threshold linker length is 50 residues in Figure 2A). It should not be forgotten here that the domains in this modelling are dimensionless, due to which there is no minimum on the curve (although there appears to be a minimum imposed by the separation between binding sites, setting a minimum to Kuhn segments). 

Therefore, spatially confined diffusional search by the free domain can result in processivity under certain circumstances, when (re)binding by the free domain is kinetically favored over dissociation of the tethering domain. Next, we asked how the flexibility of the linker affects binding time by the free domain. To this end, we ran the statistical kinetic model by varying the length of Kuhn segments (and therefore the persistence length of the chain, see Supplementary Methods) from 0.88 nm (characteristic of random coil chains) to 7.04 nm (characteristic of a polyproline II (PPII) helix), and found a marked effect (Figure 2B), with a more rigid linker providing longer binding times, making the enzyme less processive (e.g., at a length of 30 residues, the enzyme is processive with a linker of 0.88 nm, but not of 3.52 nm, Kuhn-segment length), which may be a prime factor in determining the amino acid composition and sequence conservation of processive linkers, as shown later.

As the calculated binding time is an aggregate value (integrating binding events over all substrate binding sites that can be reached by the free domain, see Supplementary Equation (S10)), we intuitively expect that processivity is increased when possible binding sites are closer to each other, i.e., there are more sites within the reach of the free domain. This is formally demonstrated by varying the spacing of sites (Figure 2C), showing that a processive enzyme can be made non-processive by moving the target sites farther away (this will depend on linker length and could actually be a tuned feature of each system). Along a similar logic, one might expect that the level of processivity is higher when target sites are spread on a two-dimensional surface, by making more sites available for binding. This is formally shown in Figure 2D, where clearly the enzyme is much more processive with a two-dimensional substrate. 

Another caveat to the model calculations is if, besides qualitatively assessing whether an enzyme is processive or not, we can draw quantitative conclusions on the level of processivity (average number of steps taken before releasing the substrate). For this, one has to note that the extent of processivity (average number of elementary steps upon engagement with the substrate) is straightforward to define, but not trivial—and is probably not unequivocal—to measure. Furthermore, being a kinetic phenomenon, it may show high stochastic fluctuations and may be very sensitive to experimental conditions. 

Nevertheless, one can infer the typical linker-length range where a particular enzyme may behave processive (say, 10–100 residues, cf. intersection of red and blue traces in Figure 2A). This inference may also suggest that linker length and the distance between substrate binding sites must have co-evolved. As an additional note, whereas preferential binding (over dissociation) follows from the kinetic setup of the system, its capacity for unidirectionality does not. As a diffusive move can equally well occur in the backward direction (Figure 1D), directionality may stem from additional mechanistic elements, such as the use of energy and/or post-translational modifications of the substrate. This may even include its degradation, such as that of extracellular matrix proteins in the case of MMP-9 [33,35] or cellulose in the case of cellulases [31,32,36]. This may hinder backward movement and result in rapid unidirectional, forward translocation (Figure 1D).

### 2.3. Multiple Examples of DLD-Type Processive Enzymes

The foregoing modelling studies show the potential for processivity encoded in the DLD arrangement of enzymes. Next, we demonstrate that there are many such enzymes in biology. Out of 47 processive enzymes of various mechanisms (Table S1), a simple literature search identified 12 processive systems that appear to rely on the DLD domain arrangement, such as MMP-9 [33,37], RNAse H1 [5], or a variety of glycohydrolases [6,31,32]. These ATP-independent enzymes enlisted in Table 1, are analyzed further.

#### 2.3.1. Structural Disorder of Linkers in Monomeric Processive Enzymes

A critical element of processivity in these DLD-type of processive enzymes is the structural disorder of the linker region connecting the binding domains, which has been experimentally demonstrated in only a few cases. For example, the cellulose-binding domain can be effectively separated from the catalytic domain of cellobiohydrolase I by limited proteolysis [38], in agreement with the extreme proteolytic sensitivity of IDPs [34]. Structural disorder was directly observed in cellulase Cel6A and Cel6B by small-angle X-ray scattering (SAXS) [39], in xylanase 10C by X-ray crystallography [40], and in MMP-9 by atomic-force microscopy (AFM) [33]. Besides these few examples, however, structural disorder has not yet been systematically analyzed in monomeric processive enzymes. 

**Figure 3:**
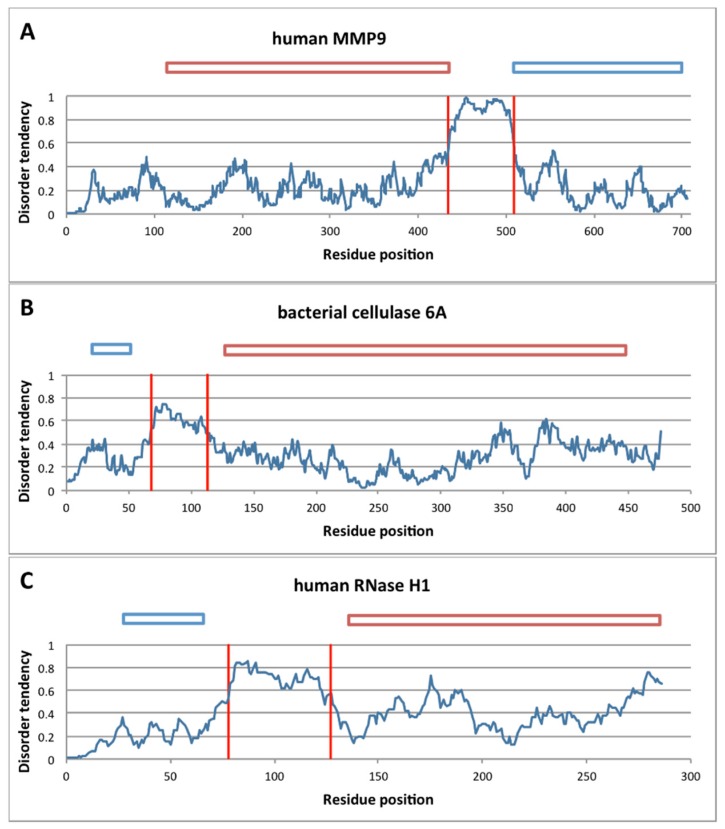
Structural disorder of linker regions in processive enzymes. The linker region in monomeric processive enzymes tends to be highly disordered, as shown here for three illustrative examples by the IUPred algorithm [41]. Traces of disorder score are given for the human and matrix metalloproteinase-9 (MMP-9) sequence (**A**), bacterial cellulase 6A (**B**) and Ribonuclease H1 (RNAseH1) (**C**). In each case, the sharp transition from order to disorder (IUPred score > 0.5) and again to order clearly delimits the linker as a disordered element connecting two globular domains. Globular domains are visualized on top of the diagrams, with blue rectangles representing binding domains and red ones representing catalytic domains.

To this end, we applied bioinformatic predictions for the local structural disorder of the linker regions of DLD enzymes in Table 1 (Figure 3). Prediction of structural disorder of three processive enzymes MMP-9, Cel6A and RNAse H1 by IUPred [41] shows a distinctive pattern of a very sharp transition from local order in the binding domains to structural disorder within the linker region. Given the reliability of disorder prediction [42], we may conclude that the linker region in processive enzymes is always disordered, as confirmed for all the cases collected from literature (cf. Table 1, predicted disorder values). Interestingly, the length of the linkers in these processive enzymes always falls within the critical range suggested by model calculations above (cf. Figure 2).

#### 2.3.2. Conservation of Sequence, Length and Dynamics of Linkers

Modelling (Figure 2) suggests that the length, structural disorder and rigidity of the linker are key elements of processive behavior, which may be in (co)evolutionary link with the typical distance between binding sites (step size) of the given system. This inference also suggests evolutionary constraints on the length and physical properties of the linker regions in these enzymes. We address this issue next.

Regarding evolutionary conservation, IDPs/IDRs have been roughly classified into three classes [43], constrained (where both sequence and structural disorder are conserved), flexible, where sequence varies but structural disorder is conserved, and non-conserved where both lack evolutionary conservation. The underlying assumption in this classification is that disordered regions that function by molecular recognition tend to have conserved sequences, whereas those having linker function are free to evolve, as long as they preserve their structural disorder. As shown in our modelling studies (Figure 2), however, spatial confinement does limit the acceptable length and flexibility of the linker. We assessed these features of the linkers for the 12 DLD-type processive enzymes in Table 1.

**Figure 4:**
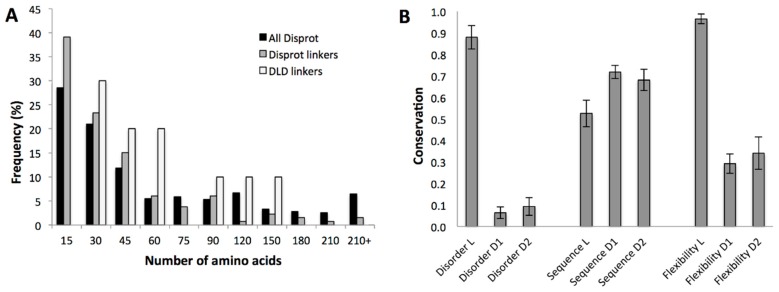
Length distribution and conservation of linker regions in DLD type processive enzymes. (**A**) Length distribution of linkers in DLD enzymes (Table 1), in comparison with that of all disordered regions and disordered linkers in the DisProt database [44]. (**B**) Comparison of the variance (mean values of the data ± SD) of structural disorder (predicted by IUPred [41]) flexibility (as approximated by the ratio of flexible residues predicted by DynaMine [45]) and sequence (assessed by DisCons [22]) of the linkers (L) and their flanking domains (D1 and D2) of the processive DLD type of enzymes (from Table 1) calculated for sequences in species given in (Table S2). Sequence conservation is defined in Section 4 Data and Methods.

**Figure 5:**
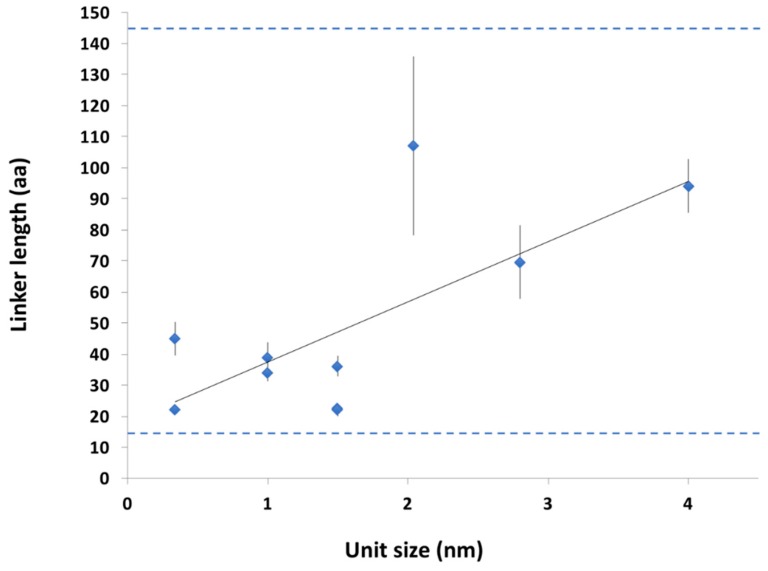
Linker length in DLD enzymes correlates with step size. Linker length in amino acids of the DLD-type processive enzymes (Table 1) is plotted as a function of the unit (step) size in the given substrate. The unit size is the size of the elementary unit (e.g., cellobiose in cellulose, nucleotides in RNA and DNA cf. Table S4) derived from the geometry of the substrate, which is the first approximation of the size of elementary steps the enzyme may take along the given substrate. The linear fit shows the correlation between the two (R^2^ = 0.4998), whereas horizontal dashed lines show the shortest and longest linker that occurs in DLD processive enzymes (Figure 4A).

In agreement with this expectation, their length shows notably narrower distribution than that of all disordered regions and all disordered linker regions in the DisProt database [44]. Processive enzymes have no short (<30 residues) or long (>150 residues) linkers, although there are many such examples of IDRs in general (Figure 4A). Furthermore, there are characteristic differences between the different DLD enzyme families (Figure S2), which also suggests a co-evolutionary relationship with the typical step size the enzyme takes. When the mean of the linker length of different families is plotted as a function of unit size of different substrates (Table S2), we can see an increase in linker length with the lengthening of processive steps (Figure 5). 

This suggests an adaptation of linker length to the geometry of the actual substrate, which also explains: (i) very similar linker length of different processive enzymes functioning on the same substrate, and (ii) the lack of very short and very long linkers in this functional class (Figure 4A and Figure 5).

Their particular function also suggests that selection pressure may also act on their flexibility. As suggested by the above classification [43], classical entropic-chain linker functions are manifested in flexible disorder, where the sequence of the disordered region is rather free to vary, but structural disorder itself is conserved; this is what is expected for the linkers of DLD-type processive enzymes. Therefore, we analyzed the evolution of these features next (Figure 4B). First, we have shown that structural disorder of DLD linkers is highly conserved (as defined in Section 4 Data and Methods), i.e., it shows very little variation. This does not necessarily entail conservation of the sequence (as suggested by flexible disorder [43]), in fact we observe that linker sequences are rather free to vary. Even though structural disorder of the linkers is conserved, it may not necessarily mean that their level of flexibility is maintained at the same level, although this is a critical feature of linkers for the level of processivity (cf. Figure 2). Actually, it was experimentally shown for a similar linker by NMR that despite extreme sequence variation, the flexibility of a linker is maintained [46]. To formally address this issue in DLD linkers, we applied the DynaMine tool developed for assessing local dynamics of IDP backbones [45]. As expected, the overall flexibility of the linker is very high and hardly varies in any of the processive enzymes (Figure 4B). 

Another characteristic closely linked with flexibility of linkers is their charge state, i.e., net charge and charge distribution, because they are among the primary determinants of the chain dimensions and conformational classes of IDPs [47], and even in the lack of hydrophobic groups, polar IDPs/IDRs may favor collapsed ensembles in water. To evaluate sequence polarity, usually the net charge per residue (NCPR), total fraction of charged residues (FCR) and the linear distribution of opposite charges (characterized by κ value) [48] are considered. Interestingly, for all the DLD linkers, their NCPR is low and their FCR is below the threshold of 0.2 (Figure S3), suggesting that they tend to have very similar behavior (they are weak polyampholytes), preferentially populate collapsed states [48]. Their low κ value (Table 1), however, suggests that they tend to have coil-like conformations. It is of note that high proline content may make the structure more extended than simply suggested by charge distribution suggests. In our case, eight out of 12 proteins have high proline content, with the exception of the two proteins in the boundary region (1: Human RNAse H1 and 5: *Clostridium cellulolyticum* Cel48F, cf. Table 1), which do not have high proline content.

#### 2.3.3. Specific Sequence Features of Processive Linkers

**Figure 6:**
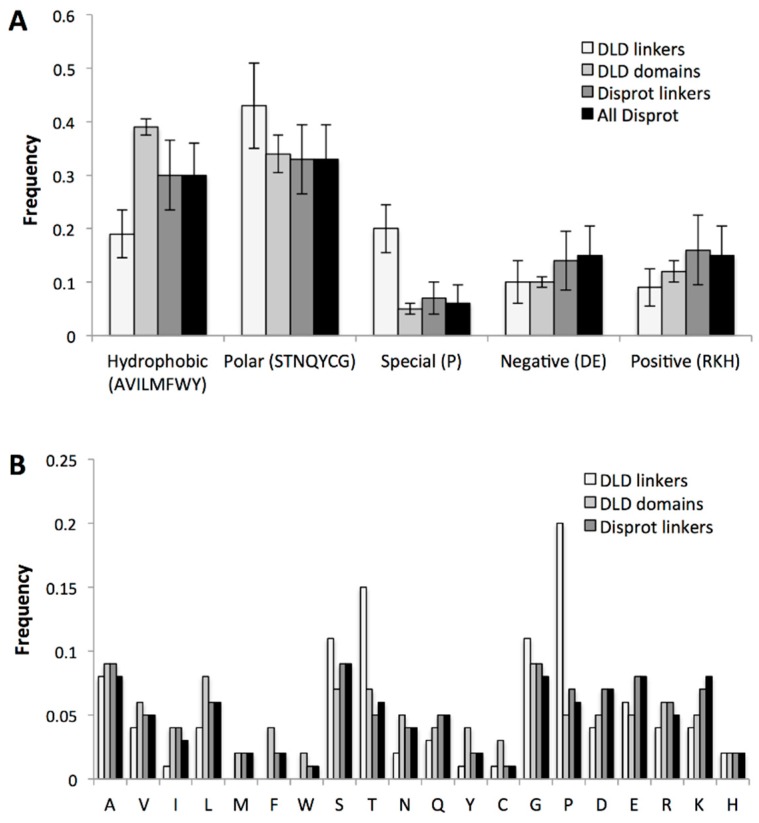
Special features of amino acid composition of linkers. Amino acid composition of linkers in DLD processive enzymes was analyzed and depicted with reference to similar measures of other data. (**A**) Amino acids of linkers were grouped into five categories and compared to the composition of non-linker (binding domain) regions of DLD enzymes (in Table 1) and also of all disordered linkers and assigned disordered linkers in the DisProt database [44]. (**B**) The abundance of amino acids in linkers and non-linker regions in DLD processive enzymes and in all disordered regions and assigned linker regions in the DisProt database.

Disordered linkers can also be classified by their amino acid composition [49]. Processive linkers in DLD enzymes may also be under special pressure in this regard, because their potential to interact with the flanking domains and/or with other protein partners, or to undergo regulatory post-translational modifications (PTMs), may be of paramount importance. To assess these features, we analyzed the amino acid composition of disordered linkers in DLD enzymes and compared them to that of DisProt linkers and all disordered regions and annotated disordered linkers in the DisProt database [44] (Figure 6). Our results show that processive linkers have significantly less hydrophobic residues than other linkers and disordered proteins in general, which suggests they have to avoid hydrophobic collapse (cf. restraints on κ value stated above) and/or interactions with partners, which most often is mediated by motifs of hydrophobic character [50]. On the other hand, they are enriched in Pro and Gly (denoted as special residues, Figure 5A only shows P under ‘special’), which entails that they have to remain extended and flexible and have a balance in oppositely-charged residues (D + E vs. R + K). Probably also for the same reason, they are, on average, more polar.

A further notable feature of DLD linkers is their enrichment in Ser and Thr, which may be indicative of frequent O-linked glycosylation and/or regulatory phosphorylation. A search in UniProt [51] for post-translational modifications (PTMs) of the DLD linkers shows several such modifications in these enzymes (Table 2).

These modifications may impact their kinetic and structural parameters and may tune their interaction with one of the domains of the flanking domains or with external partners. For example, the linker of cellulase emerges from a point not proximal to the cellulose substrate, rather from a point behind, i.e., the kinetic behavior of the enzyme is fine-tuned by the binding of the linker to the surface of the catalytic domain (see next section). Regulated linker-domain interactions are also instrumental in MMP-9, in which the linker has two short binding motifs, that bind the catalytic domain of the enzyme [35]. 

The primary function of linkers in DLD processive enzymes is to ensure relatively unrestricted spatial search of domains for binding sites along a multivalent (polymeric) substrate partner. They, however, are also often involved in the regulation of the functioning of the enzyme, as witnessed by additional binding functions and/or PTM events within the linkers themselves (for PTMs, data are either taken from UniProt or from the reference given).

#### 2.3.4. Modelling Cellulase, a Processive Enzyme

**Figure 7:**
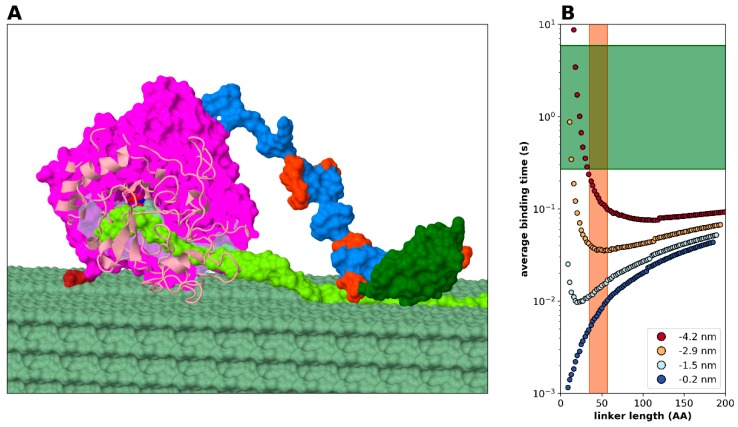
Cellulase: a model processive enzyme. (**A**) Model of the Cel7A cellulase based on the structure PDB 8cel for the catalytic domain (CD) and PDB 2mwk for the cellulose-binding domain (CBM). The CD is purple with the cellulose tunnel shown in transparent blue. One glycosylation of the CD is visible in dark red. Further elements marked are the two catalytic amino acids (red and blue stick-and-ball), the linker region (blue with orange mannose glycosylation), the CBM (dark green), and the cellulose sheet (pale green) of which one fibril (yellow-green) is being processed. The sequence and glycosylation is based on UniProt P62694. (**B**) Statistical kinetic modelling considering geometry (size) and binding of the linker to CD shows binding times characteristic of this system. The green area represents typical catalytic times for Cel7A cellulase family (Table S3), whereas the red area marks typical linker region lengths (Figure S2). The four curves correspond to various values of the linker region’s partial binding to the CD, which results in it emerging from the CD at different points (see color mark). If we consider the beginning of the CD domain as the origin of the coordinate system and the cellulose filament moves along the X axis, and assume no binding between the linker and the CD, then free end of the linker region reaches −4.2 nm (red in color scale). When the largest portion of the linker is bound to the CD, the starting point of the free linker end is at zero (blue in color scale). Yellow and light blue colors represent intermediate back-binding cases, with −2.5 and −1.5 nm starting points, respectively.

Based on all the foregoing analyses, it appears compelling that the DLD arrangement makes enzymes processive. This seems a general phenomenon, which can be demonstrated by low-resolution statistical-kinetic modelling (Figure 2). Here we proceed to show that by incorporating structural details, i.e., atomistic structural models of the domains, into the model and considering domain-linker interactions (Figure 7), we can quantitatively describe the mechanistic and kinetic behavior of one of the most-studied DLD processive enzymes, that of bacterial cellulase (*Trichoderma. reesei* Cel7A, cf. Table 1). Cel7A has two domains of different size, a larger catalytic domain (CD) that confines the linear cellulose substrate, i.e., in itself tends to be processive, and a smaller cellulose binding domain (also termed motif, CBM) attached with a disordered linker of 33 amino acids in length (Figure 7A). The enzyme is processive, typically carrying out about 20–100 cleavage events before dissociating form its substrate. By modeling all parameters of: (i) linker length and flexibility, (ii) catalytic parameters of the enzymatic domain (for the range of kinetic parameters within the Cel7A family, cf. Table S3) and binding parameters of the free (binding) domain, (iii) structural hindrance arising from the actual structures of the domains and domain-linker interaction, and (iv) distance of cellulose binding sites, we show that average binding time of the CBM domain (Figure 7B) undergoes a minimum at a linker length range that is very close to the observed linker lengths in cellulases (Table 1). Furthermore, binding of the linker to the CD has an effect on the behavior of the system (Figure 7B, cf. blue region in color scheme) as it restricts the freedom of movement of the domains, making it less processive. Since all the known cellulase linkers are highly flexible and contain little or no secondary structural elements, changing the Kuhn-segment length is not applicable in this system. The level of processivity that can be approximated as the ratio of the time of binding of CBM to the time of the catalytic reaction (for the CD of cellulase, Table S3, measured with rather artificial substrates) is on the order of 10–100, which agrees with the values reported (Table 1).

## 3. Discussion

Processivity is a basic device of enzymes working on (generating, modifying or moving along) polymeric substrates [1]. By its very molecular logic, it increases cellular economy by limiting the production of metabolic by-products and the dissipation of energy, and it enables large-scale molecular changes to occur, thus it is at the heart of many key cellular processes. Due to the all-or-none character of the operation of processive enzymes, however, there have to be very precise and highly controlled cellular mechanisms for turning them on.

As outlined, there are diverse molecular mechanisms underlying processivity, falling into two general categories, structural confinement by well-folded binding elements and spatial confinement by independent binding elements connected through a linker region. This latter mechanism is apparent in dimeric mechanochemical motors and also in monomeric enzymes. The importance of the general kinetic consequence of processivity can be deduced from its convergent appearance in many independent systems. Whereas its mechanistic underpinning is rather well understood in the case of enzymes that rely on structural confinement and is also analyzed rather extensively in the case of mechanochemical motors, it has so far been largely overlooked in the case of monomeric enzymes. 

The typical design of such enzymes is embodied by certain bacterial cellulases, which have a modular structure that combines a large CD linked to a smaller CBM by an intrinsically disordered linker [39] that enables a continuum of conformations. A similar feature has been suggested for the matrix metalloproteinase MMP-9 [33,37], which progressively degrades polymeric components of the extracellular matrix, such as collagen. This enzyme also has a modular structure, with an N-terminal unit of a catalytic domain and three fibronectin type II exosite modules, connected by a 54-residues long linker to a C-terminal hemopexin C domain. SAXS and AFM demonstrated that it can assume multiple conformations and that it can crawl in an inchworm-like manner along its substrate [57]. A similar architecture has been suggested and/or theoretically modelled in the case of glycohydrolases, such as Cel7A [58], cellobiohydrolase I [59] and chitinases [60]. The importance of this arrangement is underscored by cellobiohydrolase I, in which the deletion of the linker dramatically reduces the rate of crystalline cellulose degradation [32] and also other glycoside hydrolases, in which the removal of the carbohydrate-binding module results in a significant decrease in their activity [6], without directly affecting their catalytic domain. Apparently, the unifying feature of all these examples is the structural disorder of their linkers, which ensures a high local concentration and relatively restricted conformational search of binding domains around their binding sites. 

Here, we used statistical-kinetic modelling of such systems that this structural arrangement can endow such an enzyme with the capacity of processive movements along a polymeric substrate of spatially repeating binding sites. We characterized these enzymes by the time of (re)binding as a function of linker length, and found that within a certain length range, they have a preference for binding over dissociation, i.e., they show processive kinetic behavior. Geometric features of the domains, direct binding of the linker with the domains themselves and PTMs of the linkers all influence binding kinetics and may thus serve as points of regulatory input. This might be of no negligible importance, as the processive chain of events past the point of activation appears uncontrolled, which may have dire consequences. A proper regulatory input halting the reaction may be a remedy under some circumstances, as suggested by frequent PTMs of processive linkers (Table 2) and their regulated binding to the flanking domains, as shown for MMP-9, for example [33].

These theoretical observations have general relevance and are supported by a collection of 12 such enzymes that all have highly disordered linkers. Notably, despite rapid evolution and sequence variability of IDPs/IDRs in general, and disordered linker regions in particular, the length and flexibility of linkers in the processive enzymes is conserved. Quantitative modelling of the cellulase enzymes is in general agreement with the observed level of processivity and suggests that this functional-kinetic property is manifest in a relatively limited range of linker lengths, which appear to be in co-evolutionary link with the particular step size along their typical substrate. This has been also suggested by the behavior of the related mechanochemical motors kinesin-1 and kinesin-2, the degree of processivity of which sharply changes by changing the length of their linker regions [15]. This feature is also underlined by the observation that short and long linkers are entirely missing in DLD-type processive enzymes. 

In a broader functional context, we suggest that this observed behavior is a special case of the entropic chain functions of IDPs/IDRs and appears as a conceptual extension of mechanisms, such as fly casting [27] and monkey-bar mechanism [28]. Processivity appears to draw on all these mechanisms and may represent one of the primary benefits of the flexibility emanating from structural disorder [25,61]. This type of function cannot be supported by a structured protein; thus it is an appealing addition to the functional arsenal of structural disorder, understanding of which may even enable the design and generation of enzymes of improved capacity for the needs of biotechnology.

## 4. Data and Methods

### 4.1. Collection of Processive Enzymes and Intrinsically Disordered Proteins 

Processive enzymes were collected from the literature by searching for keywords “processive” or “processivity.” We aimed for a full coverage of all types of processive enzymes, which resulted in 47 illustrative examples (Table S1), many of which were covered previously [1]. From this collection we selected 12 monomeric enzymes, for further analysis (Table 1). Due to their dominant modular arrangement, we term these monomeric processive enzymes domain-linker-domain (DLD) type. For comparative purposes, we also downloaded 1274 IDP/IDR sequences from the DisProt database (version 7.0) and selected 133 of the IDRs annotated as “linkers” [44].

### 4.2. Statistical Kinetic Modelling of Linker Regions

To assess the statistical kinetic behavior of DLD proteins we chose the Freely Jointed Chain (FJC) model and simulated it with a Gaussian approximation [36,62]. As shown by details of the model (Supplementary Methods and Figure S1), this only causes minor deviations from the analytical solution at extreme linker lengths.

An important parameter in modelling is the stiffness of the chain that characterizes its nature of spatial distribution. In the FJC model, this is described by Kuhn segments (l_k), whose measure is two times the persistence length. In a freely moving random-coil polypeptide chain this persistence length is 0.44 nm [62], whereas in a stiff polyproline helix it is roughly an order of magnitude longer. To get the number of Kuhn segments, an amino acid chain can be simulated by calculating the contour length of the chain, l_c, divided by l_k.

It is to be noted that the approximation of a kinetic phenomenon of binding and/or dissociation is only tenable if reaching the equilibrium in spatial distribution is much faster than the event of binding and unbinding, i.e., binding/unbinding is not rate-limiting. As diffusion rates of small proteins in water are on the order of 10^−6^ cm^2^ s^−1^ [63], which is equivalent to 102 nm^2^·s^−1^, the typical μs time of the unbound (“free,” for domain definitions, cf. Figure 1D) domain equilibrating within the boundaries of the model is well below the time scale of processivity steps.

### 4.3. Assessing Structural Disorder of Linkers 

Structural disorder of processive enzymes was predicted by the IUPred algorithm [41], which is based on estimating the total pairwise inter-residue interaction energy gained upon folding of a polypeptide chain. The predictor returns a position-specific disorder score in the range 0.0–1.0, and a residue with score ≥0.5 is considered as locally disordered. To characterize the disorder tendency of domains and linkers, we calculated the ratio of disordered residues within the given region.

### 4.4. Flexibility of Linker Regions

To quantify the flexibility of linkers, we used DynaMine [45], a backbone dynamics predictor that has been trained on proteins for which NMR-based chemical shifts and experimental amide bond order parameters (S2) were available. Its score falls between 0.0 and 1.0, with a threshold 0.78 separating flexible (below) and rigid (above) regions. Residue-level DynaMine values were averaged for the entire sequence of linkers to calculate an overall measure of flexibility.

### 4.5. Charge State and Kappa Value Calculation of Linkers

The charge state of linkers was characterized by three parameters [47,48]. The net charge per residue value (NCPR) is defined as |f+ − f−|, where f+ and f− are the fractions of positively- and negatively-charged residues within the linker region, respectively. The total fraction of charged residues (FCR) is defined as (f+) + (f−). The linear distribution of opposite charges is described by the kappa (κ) parameter [48], which is the mean-square deviation of local charge asymmetry from the overall sequence charge asymmetry weighted on the maximal asymmetry allowed for a given amino-acid composition. Kappa can range from 0 (when opposite charges are evenly distributed) to 1 (when opposite charges are segregated into two clusters). Kappa has a basic influence on IDP/IDR conformation, as there appears to be an inverse correlation between the kappa value and the radius of gyration of the polypeptide chain.

### 4.6. Amino-Acid Composition and Length Distribution of Linkers

The length and amino acid composition of each processive linker (Table 1) and all IDPs/IDRs in DisProt [44] were calculated. For classification purposes, we also determined composition in terms of a reduced set of amino acid types (positive/basic: Arg, Lys; negative/acidic: Asp, Glu; polar: Ser, Thr, Cys, Gln, His, Tyr, Asn; hydrophobic: Ala, Val, Met, Trp, Phe, Leu, Ile; and special: Pro, Gly).

### 4.7. Variability and Conservation of Linker Regions

The DLD-type processive enzymes studied here contain two globular domains connected by a disordered linker. To analyze their evolutionary relatedness, we applied the MAFFT (Multiple Alignment using Fast Fourier Transform) program to generate multiple alignments [64] of the sequences from several species, anchored by the flanking ordered binding domain(s), which are highly conserved. Evolutionary conservation of a given region (either disordered or folded) was calculated by an algorithm that computes the average of genetic distances between each pair of sequences in the alignment. The details of the applied method are given in [65]. The species used for alignments and conservation analysis are listed for each protein in Table S2.

## Figures and Tables

**Table 1:** ATP-independent monomeric domain-linker-domain (DLD)-type processive enzymes.

	Protein Name	UniProt ID	ATP	Partner	Linker Length	Kappa Value (Plot Region)	Processivity
**1**	*H. sapiens* **RNAse H1**	O60930	-	RNA	50 aa (78–127)	0.254 (2)	
**2**	*H. sapiens* **XPF**	Q92889	-	DNA	22 aa (821–842)	0.187 (1)	60 nucleotides
**3**	*T. reesei* **Cel7A**	P62694	-	cellulose	33 aa (445–477)	0.503 (1)	21 catalytic steps
**4**	*H. insolens* **Cel6A**	Q9C1S9	-	cellulose	46 aa (68–113)	0.288 (1)	
**5**	*C. cellulolyticum***Cel48F** *	P37698	-	cellulose	28 aa (106–133)	0.069 (2)	
**6**	*C. thermocellum***1,4-beta-glucanase** *	Q5TIQ4	-	cellulose	103 aa (688–790)	0.238 (1)	
**7**	*H. sapiens* **Telomerase**	O14746	-	DNA	94 aa (231–324)	0.252 (1)	
**8**	*X. laevis* **XMAP215**	Q9PT63	-	tubulin	121 aa (1079–1199)	0.189 (1)	25 tubulin dimers
**9**	*H. sapiens* **Chitotriosidase-1**	Q13231	-	chitooligosaccharides	31 aa (387–417)	0.263 (1)	8.6 cleavage steps
**10**	*B. circulans* **Chitinase A1**	P20533	-	crystalline-chitin	23 aa (444–466)	0.353 (1)	
**11**	*O. sativa subsp. Japonica* **Chitinase 2**	Q7DNA1	-	chitin	17 aa (74–90)	0.848 (1)	
**12**	*H. sapiens* **MMP-9**	P14780	-	gelatine	76 aa (434–509)	0.112 (1)	

* no sufficient number of orthologous proteins.

**Table 2:** Additional functions of linkers in DLD processive enzymes. Cases where the linker was shown to bind to its adjacent domain are marked with “+”.

Enzyme	UniProt ID	PTMs	Domain Binding	Ref.
*H. sapiens* RNASEH1	O60930	Phosphorylation: S74, S76		[52]
*T. reesei* Cel7A	P62694	Glycosylation: T461, T462, T463, T462, T469, T470, T471, S473, S474	+	[53]
*H. sapiens* Telomerase	O14746	Phosphorylation: S227		[54,55,56]
*H. sapiens* Nedd4-1	P46934	Phosphorylation: S670, S742, S743, S747, Y785, S884, S888. Ubiqutination: K882		
*H. sapiens* MMP-9	P14780		+	

## Supplementary Materials

Supplementary materials can be found at https://www.mdpi.com/1422-0067/20/9/2119/s1.

## Abbreviations

AFM	atomic-force microscopy
DLD	domain-linker-domain
FJC	freely jointed chain
ID	intrinsically disordered
IDP	intrinsically disordered protein
IDR	intrinsically disordered region
MMP-9	matrix metalloproteinase-9
PTM	post-translational modification
RNAse-H1	ribonuclease H1
SAXS	small-angle X-ray scattering
