# Supplementary material for: Intrinsically Disordered Linkers Impart Processivity on Enzymes by Spatial Confinement of Binding Domains
Source: Int J Mol Sci. 2019 Apr 29;20(9):2119. doi: 10.3390/ijms20092119 (PMC6540235; doi:10.3390/ijms20092119)
Supplement: Supplementary file 1 [file ijms-20-02119-s001.pdf]

Intrinsically Disordered Linkers Impart Processivity on Enzymes by Spatial Confinement of Binding Domains

Supplementary material

Supplementary methods

Statistical-kinetic modeling of disordered linkers

To address the statistical-kinetic behavior of a domain-linker-domain (DLD) protein enabled by the linker region, we applied a Gaussian approximation of the Freely Jointed Chain (FJC) model, as described in the literature [1, 2], either for a general DLD enzyme, or for the cellulase enzyme Cel7A. According to recommendation in the literature [3] the Gaussian approximation shows only minor deviation in the distribution probability curves from the exact solution and can be used for most cases (Suppl. Figure S1). For clarity and reproducibility, we recite the major features of this modeling, which allows the computation of the spatial distribution of endpoints of a FJC.

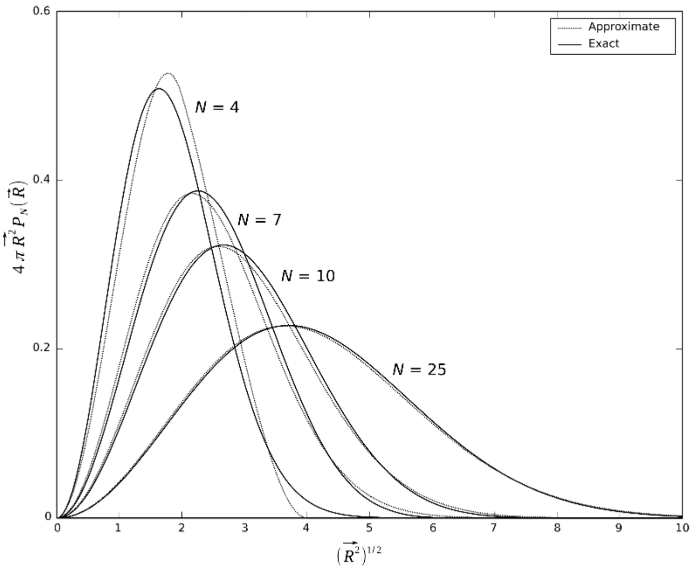

Figure S1 *Gaussian approximation of the FJC model*

Comparison between the exact solution (solid line) and the Gaussian approximation (dashed line) of the FJC model of the spatial distribution of the free binding domain around the tethered domain bound to the substrate (at x = 0). The modeling is done at different linker lengths measured in Kuhn segment units (from N = 4 to N = 25). For details, cf. ref [3].

To describe geometrical positions, let us have our Cartesian coordinate system's X axis point along a substrate chain, Y "upwards", while Z completes the XZ plane that would cover a substrate sheet.

Let $\vec{R}$ denote the end-to-end vector of the polypeptide chain, the probability density of $\vec{R}$ for an N segment chain is $\rho_N\left(\vec{R}\right)$.

$$\rho_N\left(\vec{R}\right) = \left(\frac{3}{2\pi N l_k^2}\right)^{\frac{3}{2}} exp\left(\frac{-3\vec{R}^2}{2N l_k^2}\right). \qquad \text{(Eq. S1)}$$

If needed, the chain can be calculated as 2 or more subsections as

$$\rho_{N_1,N_2}\left(\vec{R}\right) = \int \rho_{N_1}\left(\vec{R_1}\right)\rho_{N_2}\left(\vec{R}-\vec{R_1}\right). \qquad \text{(Eq. S2)}$$

The time required for computations rises exponentially with the number of segments, which rationalizes the use of the much faster gaussian approximation, despite its minor deviation from the analytical solution of FJC for linkers of very small or very large number of segments. It is to be noted that although in ATP-driven dimeric motors the description has been developed for two linkers, it is adequate for the description of a single linker in the monomeric DLD-type enzymes studied here.

As a first approximation, we may consider the two binding elements (domains in the DLD arrangement) as points with no physical extension, but in more realistic modeling we may also take into account the geometry of the protein domains, and positions of the binding sites (targets) on the substrate. While in the FJC model the individual Kuhn segments may freely overlap, we want to restrict $\vec{R}$ so that it respects the dimensions of the domains and substrate. We approximate the domains as simple spheres, and the substrate as a line or sheet with discrete binding elements.

The integral of a probability density function should always be 1.0, therefore if we exclude volumes we need to modify the function as:

$$\rho_N^* = \frac{\rho_N(\vec{R})\Theta(\vec{R})}{\int \rho_N(\vec{R})\Theta(\vec{R})d\vec{R}} \quad \text{(Eq. S3)}$$

where Θ is the exclusion function such as:

$$\Theta\left(\vec{R}\right) = \begin{cases} 0 & \text{if} \vec{R} < r_{D_1} + r_{D_2} \\ 1 & otherwise \end{cases} \quad \text{(Eq. S4)}$$

Θ can be more or less complex as the desired model requires, e.g. when a sheet-like substrate is considered.

A further refinement of the model is that part of the linker may also bind to the domain from which it originates (termed the tethering domain). In this case the probability density function changes to

$$\rho_{N_D}^* = \frac{\rho_{N_D}(\vec{R}-\vec{R_D})\Theta(\vec{R})}{\int \rho_{N_D}(\vec{R}-\vec{R_D})\Theta(\vec{R})d\vec{R}} \quad \text{(Eq. S5)}$$

Probability of docked and undocked states are denoted as $P(N_D)$, and $P(N)$ respectively. Then

$$\frac{P(N_D)}{P(N)} = e^{(-\Delta G/k_B T)} \quad \text{(Eq. S6)}$$

gives the ratio of bound and unbound states. A ΔG of $-2k_BT$, as in the case of kinesin, used as a reference, yields roughly 85% bound state. The approximation is rationalized by the notion that the amino acid composition and the length of the linker segments <u>in the DLD type enzymes are roughly comparable to the same parameters in kinesin</u>.

The probability density function then describes the local concentration of the free end of the chain as:

$$1particle * \rho_N\left(\vec{R}\right) nm^{-3} \quad \text{(Eq. S7)}$$

which can be converted to molar concentration:

$$1 particle * nm^{-3} = 1.6605 M \quad \text{(Eq.S8)}$$

Let c_N be the local molar concentration of the undocked chain, and c_{N_D} that of the docked ones, then the time of binding to a target (binding) site is:

$$t_{on} = \frac{1}{k_{on}\left(P(N)c_N + P(N_D)c_{N_D}\right)}. \quad \text{(Eq.S9)}$$

To calculate binding times for several discrete targets, we can calculate the individual local concentration at each site as $c_{N_1}, c_{N_2} \dots c_{N_n}$. Let us define $c_{N_1} \equiv c_1$ and $c_{N_{D_1}} \equiv c_1^*$. Then the aggregate binding time is:

$$t_{on} = \frac{1}{k_{on}\left(P(N)[c_1 + c_2 + c_3 \dots + c_n] + P(N_D)[c_1^* + c_2^* + c_3^* \dots + c_n^*]\right)} \quad \text{(Eq. S10)}$$

By calculating the average binding times, we can demonstrate processivity of the enzyme by showing that the free domain will find a new substrate binding site in significantly shorter time than it takes for the tethered domain to dissociate or catalyze a reaction. As (re)binding at a new binding site will in this case be preferred over dissociation, the enzyme will behave processively, and its level of processivity (the number of steps taken before falling off the substrate) can be approximated as the ratio of times of binding vs. dissociation (t_b/t_d).

Calculation of charge distribution of linkers

Charge distribution (Figure S3) of linkers was calculated using the Classification of Intrinsically Disordered Ensemble Regions (CIDER) webserver developed by the Pappu lab (http://pappulab.wustl.edu/CIDERinfo.html) [4]. The diagram is generated by the algorithm by plotting the fraction of negatively charged residues vs. the fraction of positively charged residues, giving a simple way to classify IDPs according to their conformational properties.

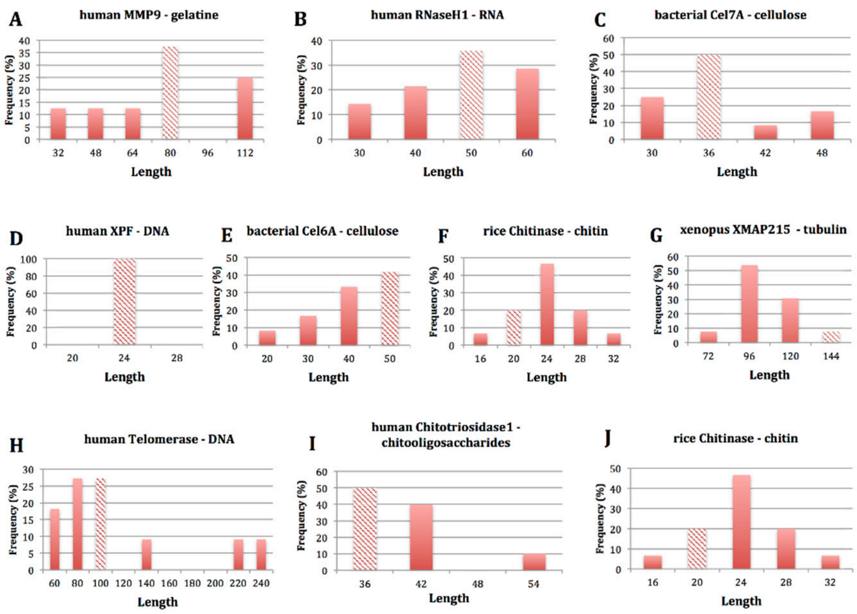

Figure S2 *Length distribution of linkers in DLD processive enzymes*

Length distribution was calculated for every DLD processive linker in Table 1, considering their homologues used for evolution and conservation studies (Suppl. Table S2). Length is shown in the number of amino acids. The striped column represent the linker length of the actual processive enzyme listed in Table 1, whereas columns of full colors give distribution of homologues.

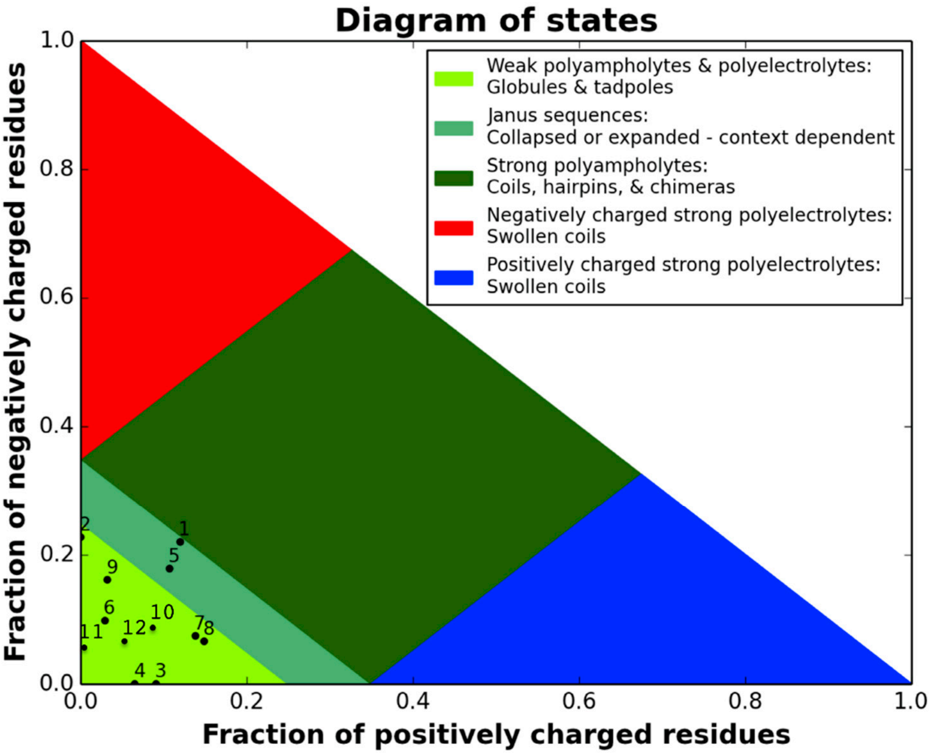

Figure S3 *Graphical representation of the charge distribution of the DLD linkers*

The charge distribution of linkers was calculated by the CIDER server as described in Suppl. methods. The light green area corresponds to weak polyampholytes or weak polyelectrolytes that form rather compact conformations. The dark green area corresponds to strong polyampholytes that form coil- or hairpin-like structures. The boundary between the two green regions represents a continuum of possibilities between these two states that lends a context-dependent nature to the sequences. Areas of blue and red correspond to either positively (blue) or negatively (red) charged strong polyampholytes that form swollen coil structures. The numbers correspond to the DLD type processive enzyme linkers in Table 1.

Table S1

Processive proteins and enzymes have been identified by text search in literature for "processive" and "processivity". In principle, the proteins can be grouped into two major categories and four substrate-categories, as follows. 1) Enzymes relying on structural confinement, such as: i) complexes with subunits that surround the substrate and ii) enzymes with asymmetric active-site cavities, and 2) enzymes relying on spatial confinement, such as: iii) dimeric mechanochemical motors and iv)

monomeric processive enzymes of domain-linker-domain (DLD) architecture. As these categories cannot always be clearly separated, they are not indicated, but important parameters relating to the possible mechanism, such as subunit structure, the presence of active-site cleft, length and disorder of linker, the measure of processivity (average number of rounds of modification/steps taken before dissociation) are given.

		Structural characteristics						
**Protein Name**	**ATP**	**Complex**	**Channel**	**Groove**	**Domain-Linker-Domain**	**Partner**	**Linker length**	**Processivity**
Yeast **40S Ribosome**	+	+	+	-	-	RNA	-	>1700 nucleotids
T. acidophilum **20S Proteasome**	+	+	+	-	-	Polypeptide	-	~140
Yeast **RNAP II**	+	+	+	-	-	dsDNA	-	1000000
**T7 gp4 helicase**	+	+	+	-	-	dsDNA	-	40000
**E1 helicase**	+	+	+	-	-	dsDNA	-	>3000 nucleotids
**T7 DNA helicase**	+	+	+	-	-	dsDNA	-	75000 bp
**T7 DNA polymerase**		dimer (gp5 and trx prot)	+	+	-	dsDNA	-	17±3 kb
Human **Upf1**	**+**	**+**	**-**	**-**	**-**	mRNA	**-**	> 10 kb
**PCNA** (in DNA polymerase δ)	-	homotrimer	+	-	-	dsDNA	-	>13000
V. Virus **Uracil DNA glycosylase**	-	3 subunit	-	+	-	dsDNA	-	1500-2000 nucleotids
E. Coli **β-protein**	+	+	+	-	-	dsDNA	-	>5000
**T4 gp45**	+	+	+	-	-	dsDNA	-	>20000
Human **Pol γ**	-	3 subunit	+	-	-	ssDNA	-	2250±162
Bacteriophage λ **exonuclease**	-	3 subunit	+	-	-	dsDNA	-	~3000
E.Coli **PNPase** (in RNA degradosome)	-	+	+	-	-	ssRNA	-	
S. antibioticus **PNPase**	-	+	+	-	-	ssRNA	-	
T. reesei **Cel7A**	-	-	-	+	+	cellulose	24 aa	20-90 acts
H. insolens **Cel6A**	-	-	-	+	+	cellulose	52 aa	
C. cellulolyticum **Cel48F**	-	-	-	+	+	cellulose	49 aa	
C. phytofermentans **Cel48**	-	-	-	+	n.a.	cellulose		3,5-6 acts

**Cellulase E4**	-	-	-			cellulose		
D. melanogaster **Kinesin-1**	+	-	-	-	+	microtubule	31 aa	1747 ± 199 n[illegible]
Mouse **Kinesin-2**	+	-	-	-	+	microtubule	17 aa	449 ± 30 nm
Neurospora crassa **Kin-3**	+	-	-	-	+	microtubule	22 aa	2.14±0.29 µm
Mouse **Dynein**	+	-	-	-	+	microtubule	204 aa	339 ± 33 nm
Gallus gallus **Myosin V**	+	-	-	-	+	actin	64 aa	2.2±0.2 µm
Human **Myosin VI**	+	-	-	-	+	actin	62 aa	796±639 nm
Xenopus **Centrosome protein E**	+	-	-	-	+	microtubule	12 aa	2.6± 0.2 µm
Human **XPF**	-	-	-	-	+	DNA	22 aa	60 nt
Sulf. solfataricus **XPF**	-	-	-	-	+	DNA	19 aa	12 nt
Staph. aureus **Helicase PcrA**	+	-	-	+	-	dsDNA	-	20
E.Coli **Exonuclease I**	-	-	+	-	-	ssDNA	-	>900
S. cerevisiae **Mip1**	-	-				ssDNA		480±20 nt
HIV **Reverse transcriptase**	-	-	-	+	-	ssDNA, ssRNA	-	<50
Human **Telomerase**	-					DNA	94 aa	
**AP-endonuclease-1**	-	-	-	+	-	dsDNA	-	200 nucl.
Human **MMP9**	-					gelatine	76 aa	
**T7 RNA polymerase**		-		+		dsDNA		thousands
Mouse **Formin** (mDia1)	-					actin	23 aa	2600 subunits
S. cerevisiae **Formin** (Bni1)	-					actin	17 aa	12000 subunits
Xenopus **XMAP215**	-					tubulin		25 tub. dimer
C. thermocellum **1,4-beta-glucanase**	-					cellulose	103 aa	
Human **Chitotriosidase-1**	-					chitooligosaccharides	31 aa	8.6±1.1
Bacillus circulans **Chitinase A1**	-					crystalline-chitin	23 aa	
Oryza sativa subsp. Japonica **Chitinase 2**	-					chitin	17 aa	

Human **Nedd4-1**	-					protein	322 aa	
Human **RNAse H1**	-					RNA	64 aa	

Table S2

Orthologues of the proteins in Table 1 were selected in different species where similar proteins were annotated. In each case the protein with highest similarity (at least 90 % homology) was chosen for analysis. Please note that for two enzymes from Table 1 (C. cellulolyticum Cel48F and C. thermocellum 1,4-beta-glucanase) are omitted because we did not found a sufficient number of homologues to carry out proper conservation analysis.

**Human MMP9**	**Human RNaseH1**	**Human XPF**	**Bacterial cellulase 7A**	**Bacterial cellulase 6A**
Homo sapiens	Homo sapiens	Homo sapiens	Hypocrea jecorina	Humicola insolens
Pan troglodytes	Pan troglodytes	Pan troglodytes	Penicillium marneffei	Corynascus sepedonium
Canis familiaris	Canis familiaris	Canis familiaris	Fusarium oxysporum	Chaetomium thermophilum
Mus musculus	Mus musculus	Bos taurus	Talaromyces stipitatus	Valsa mali
Bos taurus	Bos taurus	Mus musculus	Magnaporthiopsis poae	Nectria haematococca
Danio rerio	Gallus gallus	Gallus gallus	Neosartorya fischeri	Colletotrichum graminicola
Gallus gallus	Xenopus tropicalis	Xenopus tropicalis	Gibberella moniliformis	Trichoderma atroviride
Takifugu rubripes	Tetraodon nigroviridis	Takifugu rubripes	Aspergillus niger	Hypocrea jecorina
	Danio rerio	Danio rerio	Hypocrea virens	Trichoderma virens
	Nematostella vectensis		Necteria haematococca	Talaromyces leycettanus

	Anopheles gambiae		Gaeumannomyces graminis	Oidiodendron maius
	Caenorhabditis elegans		Gibberella zeae	Pleurotus ostreatus
	Ciona intestinalis			
	Drosophila melanogaster			

**Human Telomerase**	**Bacterial ChitinaseA1**	**Human Chitotriosidase1**	**Amphibian XMAP215**	**Rice Chitinase**
Homo sapiens	Bacillus circulans	Homo sapiens	Xenopus laevis	Oryza sativa subsp. Japonica
Canis lupus familiaris	Paenibacillus polymyxa	Pan troglodytes	Xenopus tropicalis	Ananas comosus
Pan troglodytes	Paenibacillus pabuli	Mus musculus	Homo sapiens	Citrus sinensis
Bos taurus	Paenibacillus taichungensis	Bos taurus	Pan troglodytes	Bambusa oldhamii
Mus musculus	Paenibacillus xylanexedens	Canis lupus familiaris	Gallus gallus	Daucus carota
Gallus gallus	Kurthia zopfii	Gallus gallus	Anolis carolinensis	Arachis duranensis
Anolis carolinensis	Paenibacillus tuaregi	Danio rerio	Canis familiaris	Camellia sinensis
Xenopus tropicalis	Paenibacillus barengoltzii	Xenopus tropicalis	Bos taurus	Corchorus olitorius
Takifugu rubripes	Paenibacillus rubinfantis	Takifugu rubripes	Mus musculus	Drosera adelae
Tetraodon nigroviridis	Paenibacillus senegalimassilien sis	Anolis carolinensis	Danio rerio	Hevea brasiliensis
Danio rerio	Brevibacillus brevis		Takifugu rubripes	Brassica rapa
	Brevibacillus laterosporus		Branchiostoma floridae	Vitis vinifera
			Tetraodon nigroviridis	Arabidopsis halleri
				Coffea canephora

				Pinus contorta

Table S3

Typical catalysis times of processive cellulases (which limits dissociation time of the enzyme, given in s) were collected from the literature (references are given in the main text). Parameters are given for different types of substrates (e.g. amorphous cellulose or oligosaccharide) where the corresponding values were available. CD: Catalytic domain, CBM: cellulose binding module.

**UniProt ID**	**Name**	**CD family**	**Substrate type**			
			**Amorphous Cellulose**	**Bacterial Cellulose**	**Plant Crystal-line Cellulose**	**Oligosaccharides**
**P62694**	TrCel7A	GH7	0.556 s	0.357s	9.836 s (0.2 µM Cellulose I_{α})	
					2.985 s (0.2 µM Cellulose III_{I})	
					3.209 s (0.1 µM Cellulose I_{α})	
					3.774 s (0.1 µM Cellulose III_{I})	
**P07987**	TrCel6A	GH6			0.323 s (Cellu-lose I_{α})	16.216 s (Glc3)
						0.269 s (Glc4)
**Q09431**	PcCel7D	GH7	0.5 s	0.385 s		
**A7WNT9**	ActCbh1	GH7				0.531 s (CNPLac)
						21.429 s (MULac)
**Q9C1S9**	Avi2	GH6	0.019 s (pH 8.5)			0.012 s (Cellohexaose pH 8.5)
			0.167 s (pH 9.5)			0.125 s (Cellohexaose pH 9.5)

Table S4

DLD processive enzymes move along different polymeric substrate and take various steps. The length of the elementary unit that is covered by one step of the processive enzyme is named (in

parentheses) and its typical length (unit size) is calculated from the geometry of the substrate; this length is taken as the step size for the given enzyme. The linker length distribution (mean ± SD, cf. Suppl. Figure S2) is calculated for the enzyme family (cf. Suppl. Table S2 for species considered).

**Substrate (unit)**	**Enzyme**	**Linker length (mean±SD)**	**Unit size**
RNA (nucleotide)	*Human* **RNAse H1**	44.9±10.8	0.34 nm
DNA (nucleotide)	*Human* **XPF**	22.2±0.4	0.34 nm
Cellulose (cellobiose)	*T. reesei* **Cel7A**<br>*H. insolens* **Cel6A**	33.9±5.4<br>38.6±10.3	1 nm
Telomer (hexanucleotide)	*Human* **Telomerase**	107.0±57.6	2.04 nm (0.34 nm/base pair)
Tubulin (tubulin dimer)	*Xenopus* **XMAP215**	94.1±17.3	4 nm
Chitin (trisacharid)	*Human* **Chitotriosidase-1**<br>*Bacillus circulans* **Chitinase A1**<br>*Oryza sativa subsp. Japonica* **Chitinase 2**	36.1±6.6<br>22.3±0.9<br>21.9±3.9	1.5 nm (derived from cellobiose)
Collagen (decapeptide*)	*Human* **MMP9**	69.6±23.7	2.8 nm

*for MMP9, the frequency of the consensus cleavage motif (P..HyS/T) in the substrate collagen is found to occur at about every tenth residue

References

1. Czovek, A., G.J. Szollosi, and I. Derenyi, The relevance of neck linker docking in the motility of kinesin. *Biosystems*, **2008.** *93:* 29-33.
2. Gao, D., et al., Increased enzyme binding to substrate is not necessary for more efficient cellulose hydrolysis. *Proc Natl Acad Sci U S A*, **2013.** *110:* 10922-7.
3. Bois, J., *Rudiments of polymer physics*. 2002: http://www.citeulike.org/user/norris/article/2086610.
4. Holehouse, A.S., et al., CIDER: Resources to Analyze Sequence-Ensemble Relationships of Intrinsically Disordered Proteins. *Biophys J*, **2017.** *112:* 16-21.
